# Epidemiology of Human Plague in the United States, 1900–2012

**DOI:** 10.3201/eid2101.140564

**Published:** 2015-01

**Authors:** Kiersten J. Kugeler, J. Erin Staples, Alison F. Hinckley, Kenneth L. Gage, Paul S. Mead

**Affiliations:** Centers for Disease Control and Prevention, Fort Collins, Colorado, USA

**Keywords:** Plague, epidemiology, zoonoses, autochthonous, emerging infectious diseases, bacteria, antibiotic drugs, antibacterial, antimicrobial, United States, *Yersinia pestis*

## Abstract

We summarize the characteristics of 1,006 cases of human plague occurring in the United States over 113 years, beginning with the first documented case in 1900. Three distinct eras can be identified on the basis of the frequency, nature, and geographic distribution of cases. During 1900–1925, outbreaks were common but were restricted to populous port cities. During 1926–1964, the geographic range of disease expanded rapidly, while the total number of reported cases fell. During 1965–2012, sporadic cases occurred annually, primarily in the rural Southwest. Clinical and demographic features of human illness have shifted over time as the disease has moved from crowded cities to the rural West. These shifts reflect changes in the populations at risk, the advent of antibiotics, and improved detection of more clinically indistinct forms of infection. Overall, the emergence of human plague in the United States parallels observed patterns of introduction of exotic plants and animals.

## Abstract 2

Epidemiologic changes reflect shifts in the populations at risk, the advent of effective therapy, and improved detection methods.

Plague is a globally distributed, zoonotic disease caused by the bacterium *Yersinia pestis* (*1*,*2*). In the late 1890s, rat-infested steamships introduced the disease into the continental United States (*1*,*3*,*4*). The first documented autochthonous human infection occurred in the Chinatown section of San Francisco, California, in March of 1900. Cases were soon reported in other port cities, including New Orleans, Galveston, Seattle, and Los Angeles (*3*,*5*). Along the Pacific Coast, infection spread from urban rats to native rodent species, and by the 1950s, *Y. pestis* had spread eastward to reach western portions of the Dakotas, Nebraska, Kansas, Oklahoma, and Texas. This distribution has remained static for >60 years, presumably the result of climatic and ecologic factors that limit further spread (*3*,*5*–*9*). Although poorly defined, these factors may be related to the ecology of vector species rather than that of rodent hosts (*8*).

The history of human plague in the United States provides a unique opportunity for long-term study of a zoonotic disease introduced onto a continent. Although the medical and scientific literature has detailed case histories and epidemiologic findings of plague cases in the United States, most reports have been limited in geographic scope or time frame (*4*–*6*,*10*–*21*). We use data from all reported human plague cases in the United States during 1900–2012 to summarize and describe changes in the epidemiology of plague since its introduction.

## Methods

The basis for plague diagnosis has changed over the last century. For purposes of this summary, a case of plague was defined as a clinically compatible human illness and at least 1 of the following: 1) *Y. pestis* isolated from or detected in a clinical specimen, 2) elevated antibody titer to *Y. pestis* F1 antigen in > 1 serum specimen (*22*), or 3) supportive epidemiologic and other laboratory evidence (e.g., visualization of typical *Y. pestis* morphology on a stained slide). The clinical form of plague (e.g., bubonic, pneumonic, septicemic) was determined on the basis of explicit notations in the case records or from available clinical details; only the primary clinical form was considered. For example, patients who had primary bubonic plague and secondary pneumonic plague were classified as having bubonic plague.

Data from cases occurring during 1900–1981 were collected from lists maintained by the United States Public Health Service and later the US Centers for Disease Control and Prevention (CDC), and enhanced with additional data sources including state reports and publications in the peer-reviewed literature. Supplementary detailed information on clinical course, exposure, and treatment was collected and maintained for most plague patients beginning in 1956, continuing through the present time.

Human cases acquired within the continental United States were included in this summary. Case-patients were geographically represented by state of residence or state of exposure, as indicated. Antibiotics considered effective for plague for the purpose of this analysis were: streptomycin, gentamicin, tetracycline/doxycycline, chloramphenicol, fluoroquinolones, or co-trimoxazole (*22*). Categorical variables are described as counts and proportions, and statistically compared by using χ^2^ tests with α = 0.05. Continuous variables are described by median and range.

## Results

### Descriptive Epidemiology

A total of 1,006 human plague cases were reported during 1900–2012. Infections were acquired in 18 states among residents of 20 states. Median patient age was 29 years (range <1–94 years), and 644 (65%) of the 992 patients for whom sex was reported were male. Among 913 cases with documented primary clinical form, 744 (82%) were bubonic, 74 (8%) pneumonic, 87 (10%) septicemic, 6 (1%) pharyngeal, and 2 (<1%) gastrointestinal. White non-Hispanic persons accounted for 55% of all cases when race or ethnicity was known. American Indian and Asian persons each accounted for 16%. Persons identified as Hispanic comprised 12% of cases; for 20%, race or ethnicity were unknown. Overall patterns of disease frequency and geographic distribution suggest 3 distinct epidemiologic phases over the course of the 113-year period. These phases correspond roughly to the periods 1900–1925, 1926–1964, and 1965–2012 (Figure).

**Figure:**
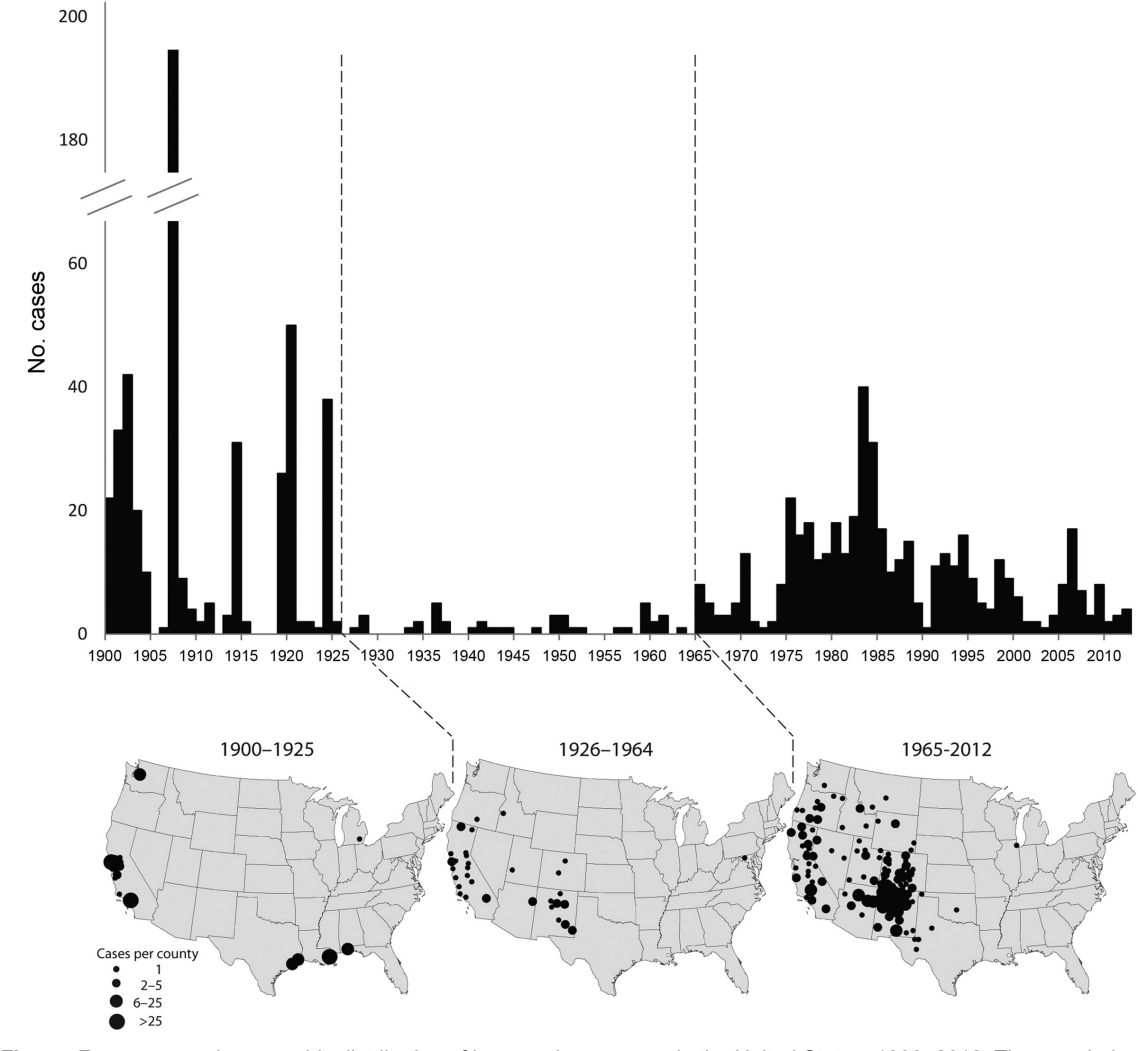
Frequency and geographic distribution of human plague cases in the United States, 1900–2012. Three periods reflect different epidemiologic and geographic patterns: 1900–1925, 1926–1964, and 1965–2012.

During 1900–1925, 496 plague cases were reported (median 3.5 cases per year), accounting for roughly half of all human plague cases in the United States during the 113 years surveyed (Table 1). Cases were restricted almost exclusively to port cities on the Pacific and Gulf coasts; 90% occurred in California and Louisiana (Figure). Variation among years was pronounced: 191 cases were recorded in 1907, and 0 were recorded in 5 (19%) of the 26 years (Table 1, Figure) in this period. The median age of patients was 30 years (range <1–84 years): 71% were male, and >30% were identified as Asian (Table 1). This period included several outbreaks of pneumonic plague characterized by person-to-person transmission; the last of these occurred in 1924 in Los Angeles. Consequently, pneumonic cases were more common (15%) than in later periods (Table 1). The seasonal distribution of cases peaked in September.

**Table 1:** Epidemiologic characteristics of human plague, United States, 1900–2012*

Characteristic	1900–1925	1926–1964	1965–2012	All years
No. cases	496	42	468	1,006
Median no. cases per year (range)	3.5 (0–191)	1 (0–5)	8 (1–40)	3 (0–191)
No. counties with reported case exposures	18	32	113	135
Years with no reported cases, %	19	44	0	20
Male sex	341/483 (71)	35/42 (83)	268/467 (57)	644/992 (65)
Median age, y (range)	30 (<1–84)	15 (3–67)	28 (<1–94)	29 (<1–94)
Race/ethnicity				
White	224/409 (55)	19/23 (83)	198/370 (54)	441/802 (55)
Asian	127/409 (31)	0/23 (0)	3/370 (1)	130/802 (16)
American Indian	1/409 (<1)	3/23 (13)	123/370 (33)	127/802 (16)
Hispanic	46/409 (11)	1/23 (4)	46/370 (12)	93/802 (12)
Black	11/409 (3)	0/23	0/370	11/802 (1)
Primary clinical form				
Bubonic	344/415 (83)	31/36 (86)	369/462 (80)	744/913 (82)
Pneumonic	60/415 (15)	3/36 (8)	11/462 (2)	74/913 (8)
Septicemic	8/415 (2)	2/36 (6)	77/462 (17)	87/913 (10)
Pharyngeal	3/415 (<1)	0/36 (0)	3/462 (1)	6/913 (1)
Gastrointestinal	0/415 (0)	0/36 (0)	2/462 (<1)	2/913 (<1)
Route of infection, no. (%)†				
Person-to-person	49 (10)	0	0	49 (5)
Known flea bite	2 (<1)	3 (7)	101 (22)	106 (11)
Animal butchering/skinning	6 (1)	8 (19)	50 (11)	64 (6)
Animal bite/scratch/cough	2 (<1)	0	19 (4)	21 (2)
Animal handling	0 (0)	5 (12)	86 (18)	91 (9)
Unknown	437 (88)	26 (62)	238 (51)	701 (70)

*Values are no. assessed/no. cases (%) except as indicated. †During 1965–2012, a total of 26 case-patients had both known flea bite and animal contact.

The second period, 1926 to 1964, accounted for only 42 (4%) of the 1,006 cases, a median of 1 case per year (Table 1). Nevertheless, the rare cases were distributed inland, and except for 1 laboratory-acquired case in Maryland, were acquired in multiple western states (Figure). Overall, 52% (22/42) of cases occurred in California, 29% (12/42) in New Mexico, and 1 or 2 cases each in Arizona, Colorado, Idaho, Oregon, and Utah. No more than 5 cases were reported in any single year (Table 1; Figure), and no cases were reported in 17 (44%) of the 38 years in this second period. The median age was markedly lower (15 years; range 3–67 years), more patients were male (83%), and more were non-Hispanic Whites (83%), as compared to other periods (Table 1). The median disease onset was in July, which was 2 months earlier than for 1900–1925.

There were 468 (47%) reported plague cases during the period of 1965–2012 (Table 1). A median of 8 cases occurred per year, and unlike previous periods, cases occurred every year for the entire 48 years (Table 1; Figure). The majority of cases occurred in the Southwest; 54% (251/467) of patients were infected in New Mexico, 14% (63/467) in Arizona, 12% (56/467) in Colorado, and 9% (42/467) in California (Figure). The median age of case-patients was 28 years (range <1–94 years). Males accounted for a lower proportion (57%) and American Indian persons represented a much larger proportion of cases (33%) than during previous years. Primary septicemic disease was more common than during earlier periods, accounting for 17% of all cases (Table 1). The median disease onset was July, the same seasonal distribution as the previous time period.

### Clinical Features and Outcome

The first documented use of antibiotics to treat plague in the United States was in 1942. Among 511 plague cases occurring before 1942 with outcome information, 336 (66%) were fatal, with similar case-fatality rates for male and female patients (Table 2). Mortality rates were highest among patients with septicemic (89%) and pneumonic (93%) forms of infection (Table 2). In addition, the mortality rate during this time was higher among racial minorities (91% overall) than among non-Hispanic Whites (53%) (Table 2).

**Table 2:** Mortality among the plague patients in the preantibiotic (1900–1941) and antibiotic (1942–2012) eras, United States

Characteristic	No. deaths/no. cases (%)		
	1900–1941		1942–2012
Total per period	336/511 (66)	75/478 (16)
Sex			
M	238/353 (67)		53/282 (19)
F	93/145 (64)		22/195 (11)
Race/ethnicity			
White	123/231 (53)		34/205 (17)
Asian	121/127 (95)		1/3 (33)
American Indian	1/1 (100)		19/122 (16)
Hispanic	38/47 (81)		6/44 (14)
Black	10/11 (91)		0
Primary clinical form			
Bubonic	235/354 (66)		47/375 (13)
Pneumonic	55/59 (93)		5/14 (36)
Septicemic	8/9 (89)		21/78 (27)
Pharyngeal	2/3 (67)		2/3 (67)
Gastrointestinal	0		0/2

After the introduction of antibiotics, the overall proportion of plague infections that resulted in death decreased substantially, from 66% to 16% (Table 2), albeit incrementally. During 1942 through 1964, 44% (11/25) of cases were fatal; after 1965, the mortality rate remained stable at ≈13%. Lower mortality was specifically seen in those patients who received effective antibiotics. Among 433 cases with treatment and outcome information, only 34 (9%) of 377 case-patients who received at least 1 dose of an effective antibiotic died, compared with 29 (52%) of 56 who received either no treatment or ineffective treatment. Primary bubonic plague case-patients more often received effective antibiotics, although the difference was not substantial (bubonic 89%; septicemic 81%; pneumonic 79%). The magnitude of decrease in deaths was similar for most clinical forms; however, the proportion of pneumonic plague cases that were fatal (36%) remained ≈3-fold higher, and of septicemic plague, ≈2-fold higher (27%), than the proportion of bubonic plague cases that were fatal (13%). Notably, although there were few cases (*6*), 67% of pharyngeal plague case-patients died, regardless of availability of effective treatment. After the introduction of antibiotics, the overall mortality rate did not differ with patient age or race, but was higher for male patients than for female patients (19% versus 11%, respectively; Table 2).

### Sources of Infection

Information on route of exposure to *Y. pestis* was documented for only 30% of cases. Of the 305 persons for whom specific exposure information was available, 106 patients had a known flea (or “insect”) bite, 91 had recently handled an animal, 64 had butchered or skinned an animal, and 21 had reported an animal bite, scratch, or cough (Table 1). Twenty-six patients who had flea bites also had a record of animal contact. Forty-nine cases occurred as the result of person-to-person transmission, most during pneumonic plague outbreaks that occurred in crowded, urban settings in the early 1900s (Table 1). Of the remaining plague cases without exposure information, an additional 139 case-patients had documented buboes in the inguinal or femoral region, a clinical finding suggestive of flea exposure (*20*).

Among case-patients who had a known flea bite, 95 cases (90%) were primary bubonic plague and 10 (9%) were primary septicemic plague. Of the 89 case-patients who had flea-acquired bubonic plague and bubo information, 59 (66%) displayed either inguinal or femoral adenopathy. Among case-patients with a history of animal contact, most cases had primary bubonic plague, but the proportion varied by type of animal contact (91% for butchering or skinning; 77% for handling; and 71% for an animal bite, scratch, or cough). Most of the case-patients with a history of an animal bite, scratch, or cough (16/21, 76%) were exposed to domestic cats. Additionally, in 6 (43%) of the 14 primary pneumonic cases that had occurred since 1924 (the last documented case of person-to-person transmission), case-patients had contact with domestic cats.

During the antibiotic era that (beginning in 1942), the mortality rate among plague case-patients with a history of animal contact (36/173, 21%) was higher than among those with only a recognized flea bite (7/80, 9%) (χ^2^ test, p = 0.018). Notably, this difference in survival was not related to receipt of effective antibiotics. Specifically, neither history of flea bite nor of animal contact was associated with receipt of effective antibiotics (χ^2^ test, p = 0.848, p = 0.499, respectively).

Records for 27 case-patients indicated a specific exposure associated with the patient’s occupation, including the only known, nonimported plague cases to occur in the eastern United States outside of port cities (i.e., Michigan, Maryland, and Illinois) (Figure). Cases occurred in the following occupational groups: 8 (30%) veterinarians, 5 (19%) persons who worked with animals (e.g., wildlife biologist or animal control personnel), 5 (19%) plague laboratory researchers, and 3 (11%) persons who conducted autopsies during the early 1900s. Since 1924, 3 (21%) of the 14 primary pneumonic plague cases occurred in persons conducting laboratory or primate research on plague. Remaining occupation-associated exposures were less direct (e.g., a geologist on a research trip, a camp counselor).

## Discussion

Invasion of a geographical location by exotic plant and animal species has been described as a multistep process involving transport, introduction, establishment, spread, and progressive impact on the ecology and human population (*23*–*25*). Multiple introductions are often necessary for an invading species to become established, and once established, a “lag period” of years to decades often ensues during which the invading species remains relatively localized. This is typically followed by a period of rapid geographic spread, ultimately resulting in increased ecologic and human effects.

Seen from the perspective of human infection, the emergence of plague in the United States is analogous to the invasion process of exotic plant and animal species. Available evidence indicates that *Y. pestis* was introduced on multiple occasions into various port cities; however, establishment appears to have been successful only in Pacific port cities (*3*,*7*). In San Francisco, infection spread from a cycle involving urban rats and their fleas into native wild ground squirrels (*Citellus* spp.) on the outskirts of the city by 1908 (*3*). In contrast, *Y. pestis* apparently never successfully established in wild rodent populations outside of Gulf coast port cities, likely a result of inhospitable ecology and early and extensive urban rat control efforts (*3*,*5*,*8*).

For the next 2 decades, the disease remained localized to port cities and surrounding areas, causing intermittent but large outbreaks. Following this lag period of minimal spread, the pathogen began dispersing rapidly eastward from the Pacific Coast (*7*). In 1935, *Y. pestis* was documented among wild rodents outside of California. By the early 1950s, plague had reached its current geographic distribution, and since 1965 has caused consistent human illness, suggesting that *Y. pestis* has become fully entrenched in enzootic cycles throughout the West (*3*). Together with their respective flea species, multiple rodents contribute to the current ecology of plague in United States, including ground squirrels, prairie dogs, wood rats, chipmunks, deer mice, and voles (*26*).

Shifts in epidemiologic features of human plague have occurred during the years following its introduction. The demographic characteristics of plague patients are inextricably linked to the geographic distribution of the organism over time and the populations at risk in those areas. For example, the disproportionate excess of men among Asian case-patients in the early years may solely represent the male-dominated Asian immigrant population in the port cities of the Pacific at the beginning of the 20th century (*27*,*28*). The demographic shift in burden of illness from persons of Asian heritage to those of American Indian heritage over time reflects change in the geographic distribution of plague from overcrowded urban neighborhoods dominated by immigrant populations along the Pacific Coast to the rural areas in the Southwest, including tribal lands. The proportional increase over time in primary septicemic cases was likely the result of better recognition of a clinically indistinct form of infection coincident with improved laboratory diagnostic capability (i.e., blood cultures), or possibly a increase in number of infections associated with direct animal contact.

The overall plague mortality rate decreased with availability of effective treatment. Nevertheless, the risk of death from plague infection is still substantial, particularly for patients with primary septicemic, pneumonic, and pharyngeal manifestations. Although exposure information was limited, infection acquired through animal contact was associated with higher mortality than was infection transmitted by flea bite. Receipt of effective antibiotics was similar between case-patients who had animal contact and those bitten by fleas. The difference in mortality rates may be related to higher or more direct initial inoculum of bacteria or differences in protein expression between mammals and fleas (*29*–*31*).

This summary of human plague in the United States may underrepresent some infections. Although *Y. pestis* is among the most pathogenic bacteria known, and mild, self-limiting infection is not considered commonplace, this summary could exclude mild and undiagnosed infections (*32*). Unrecorded plague infections may also have occurred with unrecognized septicemic disease or among racial or ethnic minorities, whose populations typically have less access to medical care. Additionally, minorities may not have sought medical care because of fear of racial prejudice associated with the initial introduction of plague (*3*,*33*). Unrecorded plague infections would more likely have occurred in the early 20th century, however, and are unlikely to dramatically affect the overall trends observed in this analysis. 

Now an endemic zoonosis in the United States, plague is likely to continue causing rare but severe human illness in western states. Historically, plague was often linked to poor sanitation that resulted in rodent infestations. However, plague in New Mexico has increasingly occurred in more affluent areas, a result of continued suburban and exurban development in enzootic plague foci (*11*,*36*). Regardless of a person’s race, ethnicity, or socioeconomic status, the primary risk factors for plague infection in the United States are behaviors and conditions that increase both direct and indirect human contact with rodents and their fleas. In recent years, few patients have reported clear exposures to infected animals or rodent fleas; most were likely infected while performing common outdoor peridomestic work (e.g., cutting brush or chopping wood) or as a result of contact with infected fleas that were brought into the home by indoor/outdoor pets (*18*,*37*; CDC, unpub. data)*.* Clinical suspicion remains critical to early and appropriate treatment. Recommended treatment is with aminoglycosides and tetracyclines, but fluoroquinolones may also be effective (*34*). The US Food and Drug Administration recently approved levofloxacin for treatment of patients with plague, based on in vitro and animal studies (*35*). Additional antibiotics considered effective for the purposes of this summary should not be considered first-line treatment for plague. Accurate plague diagnosis is further challenged by reliance on automated identification systems that frequently misidentify *Y. pestis* (*38*). A few travel-associated cases have been diagnosed in areas without endemic plague (e.g., South Carolina, New York City, Connecticut), highlighting the importance of plague in the differential diagnosis of ill persons, even those lacking apparent lymphadenopathy, with recent travel history anywhere in the western United States (*39*,*40*; CDC, unpub. data).
